# Protective Effect of *Baccharis trimera* Extract on Acute Hepatic Injury in a Model of Inflammation Induced by Acetaminophen

**DOI:** 10.1155/2014/196598

**Published:** 2014-11-12

**Authors:** Bruno da Cruz Pádua, Joamyr Victor Rossoni Júnior, Cíntia Lopes de Brito Magalhães, Míriam Martins Chaves, Marcelo Eustáquio Silva, Maria Lucia Pedrosa, Gustavo Henrique Bianco de Souza, Geraldo Célio Brandão, Ivanildes Vasconcelos Rodrigues, Wanderson Geraldo Lima, Daniela Caldeira Costa

**Affiliations:** ^1^Programa de Pós-graduação em Ciências Biológicas do Núcleo de Pesquisas em Ciências Biológicas (NUPEB), Universidade Federal de Ouro Preto (UFOP), 35.400-000 Ouro Preto, MG, Brazil; ^2^Centro Federal de Educação Tecnológica de Minas Gerais (CEFET/MG), 35.790-970 Curvelo, MG, Brazil; ^3^Departamento de Ciências Biológicas (DECBI), Instituto de Ciências Exatas e Biológicas, Universidade Federal de Ouro Preto (UFOP), 35.400-000 Ouro Preto, MG, Brazil; ^4^Departamento de Bioquímica e Imunologia, Instituto de Ciências Biológicas, Universidade Federal de Minas Gerais (UFMG), Cx. Postal 486, 30.161-970 Belo Horizonte, MG, Brazil; ^5^Departamento de Alimentos, Escola de Nutrição, Universidade Federal de Ouro Preto (UFOP), 35.400-000 Ouro Preto, MG, Brazil; ^6^Programa de Pós-graduação em Ciências Farmacêuticas (CIPHARMA), Escola de Farmácia, Universidade Federal de Ouro Preto (UFOP), 35.400-000 Ouro Preto, MG, Brazil; ^7^Núcleo de Pesquisas em Produtos Naturais e Sintéticos, Departamento de Física e Química, Faculdade de Ciências Farmacêuticas de Ribeirao Preto, Universidade de São Paulo (USP), 14040-903 São Paulo, SP, Brazil

## Abstract

*Background*. Acetaminophen (APAP) is a commonly used analgesic and antipyretic. When administered in high doses, APAP is a clinical problem in the US and Europe, often resulting in severe liver injury and potentially acute liver failure. Studies have demonstrated that antioxidants and anti-inflammatory agents effectively protect against the acute hepatotoxicity induced by APAP overdose. *Methods*. The present study attempted to investigate the protective effect of *B. trimera* against APAP-induced hepatic damage in rats. The liver-function markers ALT and AST, biomarkers of oxidative stress, antioxidant parameters, and histopathological changes were examined. *Results*. The pretreatment with *B. trimera* attenuated serum activities of ALT and AST that were enhanced by administration of APAP. Furthermore, pretreatment with the extract decreases the activity of the enzyme SOD and increases the activity of catalase and the concentration of total glutathione. Histopathological analysis confirmed the alleviation of liver damage and reduced lesions caused by APAP. *Conclusions*. The hepatoprotective action of *B. trimera* extract may rely on its effect on reducing the oxidative stress caused by APAP-induced hepatic damage in a rat model. *General Significance*. These results make the extract of *B. trimera* a potential candidate drug capable of protecting the liver against damage caused by APAP overdose.

## 1. Introduction

The liver is the major site of detoxification and the primary target of drug exposure in the body [1]; therefore, drug-induced liver injury is a significant public health problem, accounting for over half of all cases of acute liver failure [2].

Acetaminophen (APAP) is a commonly used analgesic and antipyretic agent. At therapeutic doses, it is usually safe and well tolerated. However, acute acetaminophen overdose causes severe and fatal hepatotoxicity [3]. Some individuals experience APAP toxicity even at therapeutic doses of less than 4 g/day, and, in pediatric populations, the majority of APAP overdoses are unintentional [4]. Moling et al. [5] reported a case of severe hepatotoxicity after therapeutic doses of APAP in an adult man. In fact, APAP overdose is the most common cause of drug-induced acute liver failure in the United Kingdom and the United States [6, 7].

The toxicity of APAP is related to its bioactivation by cytochrome P450 to the electrophilic metabolite N-acetyl benzoquinone imine (NAPQI). At therapeutic doses, NAPQI is efficiently detoxified by glutathione (GSH) and eliminated through urine or bile; however, at supratherapeutic doses, both the glucuronidation and sulfation pathways become saturated, and extensive bioactivation of APAP depletes the hepatic GSH pool and causes oxidative stress [8]. This oxidative stress may trigger signaling pathways to act through mitochondrial toxicity, ultimately causing cell death [1]. A significant amount of evidence has pointed to the potential involvement of oxidative stress in acetaminophen toxicity [9, 10].

Several studies have shown that antioxidants and anti-inflammatory agents effectively protect against the acute hepatotoxicity induced by acetaminophen overdose [11–13].

Several herbal medicines, their active constituents and formulations, are used in the treatment of a wide variety of clinical diseases and provide benefit to societies. Their protective action is through antioxidant enzymes (e.g., SOD, CAT, GST, and GR), which modify many pathways and proteins, including the DNA damage/repair processes [14], Nrf-2 [15], and xenobiotic response elements [16], thus maintaining the prooxidant/antioxidant balance in the body [1].

In this context, we highlight* Baccharis trimera*, popularly known as “carqueja,” a member of the Asteraceae family and a native shrub from South Brazil, Paraguay, Uruguay, and Argentina. Medicinal teas prepared from the aerial parts of this shrub are used in folk medicine to treat not only gastrointestinal and liver diseases but also inflammatory processes [17]. Because of these biological effects, research on the chemical composition of* B. trimera* was conducted and demonstrated that this plant has many bioactive compounds, such as flavonoids, diterpenes, and triterpenes [18]. Triterpenes have been reported to be primarily responsible for its anti-inflammatory activity [19, 20], while the flavonoids, due to their antioxidant activity, have been linked to protecting the body against reactive oxygen species (ROS) [21].

This study was conducted to investigate the effect of* B. trimera* in the modulation of oxidative stress and to evaluate the preventive effect of* B. trimera* in acetaminophen-induced liver damage.

## 2. Materials and Methods

### 2.1. Reagents

The chemical reagents, including DTNB [5,5′-dithio-bis (2-nitrobenzoic acid)], 2,4-dinitrophenylhydrazine (DNP), and thiobarbituric acid (TBA), were purchased from Sigma-Aldrich (St. Louis, MO, USA). Acetaminophen (APAP) (200 mg/mL) was obtained from Janssen-Cilag Pharmaceuticals, Brazil. The kit for measuring serum alanine aminotransferase (ALT) and aspartate aminotransferase (AST) was from Diagnostic Labtest, Brazil.

### 2.2. Collection of Plant Material

The aerial parts of* B. trimera *were collected during August 2011 in the city of Ouro Preto, Minas Gerais, Brazil. The specimen, voucher number OUPR 22.127, was identified by Professor Viviane R. Scanlon and deposited in the Herbarium José Badini, UFOP.

### 2.3. Preparation of Extract

The aerial parts of the plant were dried in a ventilated oven, sprayed in a mechanical mill, and stored in plastic bottles. To obtain the hydroethanolic extract, approximately 100 g of the plant was extracted with distilled water and 70% alcohol at a ratio of 1 : 1 for 24 h. Vacuum filtration and evaporation of the solvent in a rotovap were then performed. The crude extract that formed, with a percentage yield of 8-9%, was then diluted with phosphate-buffered saline (PBS, pH 7.4); a concentration of 600 mg/kg body weight was used* in vivo*. The methodology for the extract preparation was based on the work of Grance et al. [22] with some modifications.

### 2.4. LC-DAD-ESI-MS Analyses

**Figure 1:**
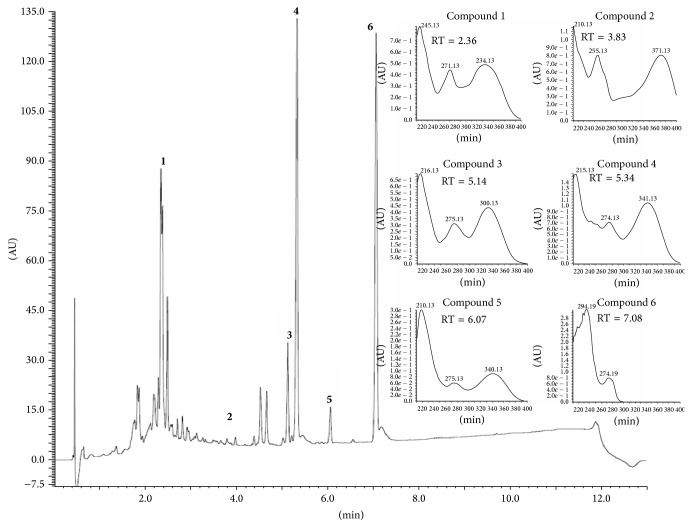
RP-UPLC-DAD profiles of hydroethanolic extract of* B. trimera*. Conditions: CHS130 100 RP-18 column (1.7 *μ*m, 50 × 3 mm i.d.). Elution was carried out with a linear gradient of water 0.1% formic acid (A) and acetonitrile 0.1% formic acid (B) (from 5% to 95% of B in 11 min) and the UPLC fingerprints were registered on a ACQUITY Waters apparatus with a UV-DAD detector (Waters 2996). Operating parameters of the mass spectrometer were capillary temperature 320°C; spray needle voltage set at 3.50 kV; ES capillary voltage +3 and −47 V for positive and negative polarity, respectively; and tube lens offset 0 and −25 V for positive and negative polarity, respectively. Nitrogen was used as a sheath gas with a flow of 50 arbitrary units. Mass analysis was carried out in full-scan mode from 100 to 1.500 amu, in both positive and negative mode. UV spectra (190–450 nm) from the main peaks are shown inside on the chromatogram.

Analyses were performed using an UPLC Acquity (Waters) ion trap mass spectrometer equipped with an atmospheric pressure chemical ionization (APCI) interface operated in the following conditions: positive and negative ion mode; capillary voltage, 3500 V; capillary temperature, 320°C; source voltage, 5 kV; vaporizer temperature, 320°C; corona needle current, 5 mA; and sheath gas, nitrogen, 27 psi. Analyses were run in the full scan mode (100–2000 u). The ESI-MS/MS analyses were additionally performed in an UPLC Acquity (Waters) with helium as the collision gas, and the collision energy was set at 30 eV. Chromatographic separation was done on ACQUITY UPLC BEH (1.7 *μ*m, 50 × 2 mm i.d.) (Waters). The mobile phase consisted of water 0.1% formic acid (solvent A) and acetonitrile 0.1% formic acid (solvent B). The elution protocol was 0–11 min, linear gradient from 5% to 95% B. The flow rate was 0.3 mL min^−1^, and the sample injection volume was 4.0 *μ*L. The UV spectra were registered from 190 to 450 nm. Mass spectrometry analysis was performed on quadrupole instrument fitted with an electrospray source in the negative mode (Figure 1). Ion spray voltage: −4 kV; orifice voltage: −60 V.

### 2.5. *In Vitro* Test: Cell Culture

The cell strains were acquired at the Cell Bank, Federal University of Rio de Janeiro (UFRJ). Liver cells were cultured in 75 cm^2^ growth vials (SARDEST) containing MEM culture medium. HEPES, 10% (v/v) bovine fetal serum, and 1% (v/v) of a mix of penicillin (200 U/mL) and streptomycin (200 *μ*g/mL) were added to the medium. The vials were stored in oven at 37°C and humidified with 5% carbon dioxide (CO_2_). This medium was replaced every two or three days depending on the confluence of the cell monolayer and the subcultures (passages) carried out. When the vials reached 100% confluence, the medium was vacuumed, and the cell monolayer was rinsed twice with phosphate-buffered saline solution without calcium and without magnesium (PBS). Later, to detach the monolayers, a solution of 0.20% trypsin and 0.02% EDTA was used. Next, the cell count was carried out with Trypan blue 0.3% in a Neubauer chamber.

### 2.6. Toxicity Assay with MTT

The cell line was acquired from the Cell Bank, Federal University of Rio de Janeiro (UFRJ). We added 5.0 × 10^3^ HEPG2 in culture medium MEM (10% v/v bovine fetal serum and 1% v/v mixture of penicillin/streptomycin). The plates were incubated in a humidified chamber with 5% CO_2_ at 37°C for 1–72 hours. After this time, the supernatant was removed, and 20 *μ*L MTT was added and incubated for 30 minutes. The absorbance was read at 570 nm in a microplate reader (Thermo Plate). HEPG2 was incubated in the presence of 1, 2, and 3% (50 *μ*L) of the* B. trimera* extract for 1 and 24 hours to assess cell viability. The calculation used to assess the percentage of cell viability was (absorbance of treated cells/absorbance control) × 100. The control was assigned 100% viability.

### 2.7. *In Vivo* Test: Animals

The Laboratory of Experimental Nutrition of the Federal University of Ouro Preto (UFOP) provided the male albino Fischer rats used in the experiment; the animals were approximately 12 weeks old and weighed approximately 180 g. All animals were kept in individual cages placed in an environment with controlled temperature, light, and humidity, and the animals received both commercial rat chow and water* ad libitum*. This work was conducted in accordance with the international standards of animal protection and with the ethical principles of the Brazilian College of Animal Experimentation, and the protocols were approved by the Ethics Committee on Animal Use (CEUA) of UFOP (OF 166/2011 protocol 2011/82).

For the experiments, thirty-two rats were divided into four groups according to their treatment. The control group (C) received 1.0 mL PBS, the* B. trimera* (Bt) group received 600 mg/kg of the* B. trimera* extract, the acetaminophen (APAP) group received a single dose of 835 mg/kg acetaminophen, and the* B. trimera* + acetaminophen (Bt + APAP) group received 600 mg/kg of the* B. trimera* extract and a single dose of 835 mg/kg acetaminophen an hour later. All treatments were administered by gavage and the interval between the* B. trimera* pre-treatments and APAP dose were based on the work of Meotti et al. [23] and Ajith et al. [24]. The animals were anesthetized and euthanized twenty-four hours after the APAP dose. The dose of APAP used and the experimental lining were based on the work of Yen et al. [25]. The animals that received 835 mg/kg APAP orally demonstrated, by the kit LabTest, a higher activity of ALT and AST. This increase in activity reached a peak 24 hours after the administration of APAP.

### 2.8. Preparation of Liver Tissue

The liver tissue was collected immediately after euthanasia of animals. To determine carbonyl protein concentrations, 200 mg of tissue was homogenized in 50 mM phosphate buffer (pH 6.7) and 1 mM EDTA. To determine the concentration of thiobarbituric acid reactive substances (TBARS), catalase and superoxide dismutase activity, 100 mg of liver tissue was homogenized in phosphate buffer (pH 7.4). Similarly, to determine total glutathione concentration, 100 mg of liver tissue was homogenized in 5% sulfosalicylic buffer. After homogenization, the samples were centrifuged at 10.000 g for 10 minutes at 4°C. The supernatant was collected and used as the biological sample.

### 2.9. Determination of Oxidative Stress Markers

#### 2.9.1. Thiobarbituric Acid Reactive Substances (TBARS)

The TBARS concentration was determined based on thiobarbituric acid (TBA) binding to oxidized lipids. This measurement was performed according to Buege and Aust [26].

#### 2.9.2. Carbonylated Protein

Protein oxidation by ROS leads to the formation of carbonyl derivatives, which can be measured by sensitive methods. Methods that use 2,4-dinitrophenylhydrazine (DNPH), which reacts with carbonyl groups to generate the corresponding hydrazone and can then be analyzed spectrophotometrically, are especially useful. Measurements of carbonylated protein were performed according to Levine et al. [27].

### 2.10. Determination of Antioxidant Defenses

#### 2.10.1. Superoxide Dismutase Activity (SOD)

The activity of total superoxide dismutase (SOD) was measured using a kit (Cayman Chemical Company, MI, USA). Briefly, hepatic tissue was homogenized in cold 20 mM HEPES (pH 7.2) containing 1 mM EGTA, 210 mM mannitol, and 70 mM sucrose. Ten microliters of the supernatant was used in the test. The reaction was initiated by adding xanthine oxidase. The plate was incubated on a shaker for 20 min at room temperature, and the absorbance was measured at 450 nm using a plate reader (Biotek ELx808).

#### 2.10.2. Catalase (CAT)

Catalase activity was determined based on its ability to convert hydrogen peroxide (H_2_O_2_) into water and molecular oxygen. The assays were performed as described by Aebi [28].

#### 2.10.3. Total Glutathione Concentration

Glutathione is present in cells mainly in its reduced form (GSH), which represents approximately 90% of the total glutathione in the cell. The remaining amount is in the form of oxidized glutathione (GSSG). To determine the levels of total glutathione (GSG + GSSG) in our biological samples, we used a Sigma kit that employs a kinetic method based on the reduction of DTNB [5,5′-dithiobis(2-nitrobenzoic acid)] to TNB, which can be spectrophotometrically measured at 412 nm. A solution of reduced glutathione (G4251-Sigma) was used to determine the standard curve. Total glutathione is expressed in nmoles per mL of sample.

#### 2.10.4. Glutathione Peroxidase Activity (GPx)

The Glutathione Peroxidase Cellular Activity Assay kit (Sigma-Aldrich, St. Louis, MO, USA) was used to measure glutathione peroxidase activity in tissue extracts. The decrease in NADPH absorbance measured at 340 nm during the oxidation of NADPH to NADP is indicative of glutathione peroxidase activity because the enzyme is the rate-limiting factor of the coupled reactions. The enzyme activity is expressed as units/mL.

#### 2.10.5. Glutathione Reductase Activity (GR)

Glutathione reductase (GR) activity was measured using a kit (Sigma-Aldrich, St. Louis, MO, USA). Glutathione reductase is essential for the glutathione redox cycle to maintain adequate levels of reduced cellular GSH, which serves as an antioxidant that reacts with free radicals and organic peroxides. The activity of glutathione reductase was measured by following the increase in absorption caused by the reduction of DTNB at 412 nm. The enzyme activity is expressed as units/mL.

### 2.11. Real-Time Quantitative RT-PCR Assay

The total RNA was extracted from 50 mg tissue using Trizol reagent (Invitrogen Life Technologies, CA, USA) according to the manufacturer's protocol and resuspended in 30 *μ*L RNase-free water. The concentration and purity of RNA were estimated spectrophotometrically from the A260/A280 ratio (NanoVue, GE Healthcare, UK). A total of 1 *μ*g RNA was converted to cDNA using oligo (dT) and a High-Capacity cDNA Reverse Transcription Kit (Applied Biosystems, Foster City, CA, USA) according to the manufacturer's recommendations.

Quantitative real-time PCR (qPCR) was performed using the* Power* SYBR Green PCR Master Mix reagent (Applied Biosystems, Foster City, CA, USA) in a final reaction volume of 12 *μ*L. The reaction included 0.1 *μ*g cDNA and 0.5 *μ*L of each primer (forward and reverse, 10 *μ*M). The forward and reverse primer sequences for Zn-SOD, Mn-SOD, CAT, glutathione peroxidase (GPx), and gamma-glutamylcysteine (*δ*-GCS) were obtained from published nucleotide sequences [29]. The reactions were performed using the ABI Prism 7300 Sequence Detector (Applied Biosystems) under the following conditions: 50°C for 2 min, 95°C for 10 min and 40 cycles of 95°C for 15 s, and 60°C for 1 min.

The specificity of the products obtained was confirmed by analysis of the dissociation curves of the amplified product. As an internal control, the expression of the housekeeping gene 18S was used. The data obtained were analyzed using the comparative C_T_ method. All analyses were performed in triplicate.

### 2.12. Histological Evaluation

Liver fragments not exceeding 4 mm in diameter were fixed in 10% formaldehyde solution and then dehydrated, diaphanized, and embedded in paraffin. Paraffin sections of approximately 4 *μ*m were obtained by sectioning embedded fragments on a rotary microtome. The sections were mounted on cleaned and degreased glass slides. The slides were stained with hematoxylin and eosin for visualization of histological damage.

### 2.13. Statistical Analysis

The data are expressed as the mean ± standard deviation (SD). All data were subjected to a normality test. After determining that the data were normally distributed, we chose to use Student's *t* test. A *P* value <0.05 was considered significant. The tests were performed using GraphPad Prism version 4.00 for Windows (San Diego, CA, USA).

## 3. Results

### 3.1. LC-DAD-ESI-MS Analysis of Hydroethanolic Extract

In hydroethanolic extract of* B. trimera* we identify five flavonoids by LC-DAD-ESI-MS obtained of chromatogram in LC-DAD, as shown in Table 1.

### 3.2. *In Vitro* Assays: Toxicity of the Hydroethanolic Extract of* B. trimera* in Liver Cells (HEPG2)

After incubation for 1 and 24 hours (Table 2), it was observed that cells treated with the extract maintained high viability of 89.0% and 98.6%, respectively, without exhibiting significant toxicity.

### 3.3. *In Vivo *Assays: Effect of* B. trimera* on Biomarkers of Hepatocellular Damage

Figure 3 shows the activity of serum transaminases in the different treatment groups. The ALT and AST transaminases were significantly enhanced (*P* < 0.05) by 9.8- and 6.3-fold, respectively, in APAP-intoxicated animals. Treatment with* B. trimera* significantly inhibited the elevation of the activity of serum transaminases ALT and AST, which was found to be 5.0- and 3.6-fold lower than that in the APAP-intoxicated animals, respectively.

### 3.4. Effect of* B. trimera* on APAP-Induced Oxidative Damage

We evaluated the oxidative damage to proteins and lipids by measuring oxidative stress markers in hepatic tissue. Specifically, we analyzed carbonylated protein and TBARS in this tissue. The results shown in Figure 4 indicate a significant increase in the concentration of carbonylated protein in the livers of animals intoxicated with APAP compared to the nonintoxicated control group. However, treatment of APAP-intoxicated rats with* B. trimera* resulted in the reduction of hepatic carbonylated protein concentration compared to the untreated* B. trimera* group. Moreover, we observed increased lipid peroxidation in rats intoxicated with APAP compared to nonintoxicated animals, and* B. trimera* treatment was also capable of reducing hepatic lipid peroxidation compared to the untreated group. Thus, these results demonstrate that treatment with* B. trimera* is capable of minimizing oxidative damage.

### 3.5. Effect of* B. trimera* on Antioxidant Status

To investigate the involvement of antioxidant enzymes in mediating the radical-scavenging activity of* B. trimera,* the mRNA levels and activities of intracellular antioxidant enzymes were measured in the different groups. The mRNA levels and activities of SOD and CAT are shown in Figure 5.

SOD and CAT function coordinately to remove superoxide radicals from the cellular system. The APAP-intoxicated group showed an increase in Zn-SOD (Figure 5(a)) and Mn-SOD (Figure 5(b)) expression compared to control animals. This increase in the expression of different isoforms of SOD was accompanied by an increase in the total activity of this enzyme in the group intoxicated with APAP (Figure 5(d)) (0.33 ± 0.07 units/mg protein) compared to the control (0.24 ± 0.036 units/mg protein). In the APAP-intoxicated group treated with* B. trimera*, the mRNA level and activity were significantly lower than their respective control (APAP group). In this study, the mRNA levels and activity of catalase decreased significantly (*P* < 0.05) in the APAP-treated rats, and this decrease in expression and activity was prevented in the group intoxicated with APAP and treated with* B. trimera*.

Figure 6 shows that there was a significant increase in *δ*-GCS (Figure 6(a)) gene expression in the livers of animals intoxicated with APAP. Treatment with* B. trimera* was able to reverse this profile, decreasing the expression of *δ*-GCS. Despite the increased expression of *δ*-GCS, there was a decrease in the concentration of total glutathione in the livers of animals intoxicated with APAP. However, treatment with* B. trimera* was able to reverse this decrease (Figure 6(b)).

The results in Figure 7 show a decrease in the mRNA and enzyme activity of GPx (Figures 7(a) and 7(b)) and in the activity of the enzyme GR (Figure 7(c)) in the livers of APAP-intoxicated rats compared to control rats.* B. trimera* was able to increase the mRNA levels of GPx and GR activities. No change was observed in the activity of GPx and GR in the livers of rats that received only the extract.

### 3.6. Histopathology

Microscopic observations revealed normal histology with regular morphology of the liver tissue in the control (Figure 8(a)) and* B. trimera* groups (Figure 8(b)). In the APAP-intoxicated group (Figure 8(c)), cellular damage was visible in the form of hydropic degeneration, inflammation, and hemorrhage. The* B. trimera* treatment (Figure 8(d)) considerably improved the liver morphology in comparison to the APAP-intoxicated rats.

## 4. Discussion

Research aiming to propose new strategies of therapeutic intervention increasingly includes the use of plant extracts and other natural products. Studies carried out in our lab have demonstrated the beneficial potential of the hydroalcoholic extract of* B. trimera* on the toxicity induced by APAP [30]. In the peripheral neutrophils of rats intoxicated with this drug, the extract was able to regulate the production of reactive oxygen species (ROS) and reactive nitrogen species (RNS). Williams et al. (2014) studied the activation of neutrophils during APAP-induced hepatotoxicity and demonstrated that ROS generation mediated by NADPH oxidase is not a critical event during liver injury caused by this drug. However, they also found that neutrophils present in peripheral blood are activated after administration of APAP. Although the contribution of inflammatory cells to the hepatic damage induced by APAP is still controversial [31], the innate immune response and the production of reactive species from different sources are correlated with many diseases that affect the liver. For example, neutrophils are associated with hepatic injury during ischemia, endotoxemia, and obstructive cholestasis in animal models [9, 32].

**Figure 2:**
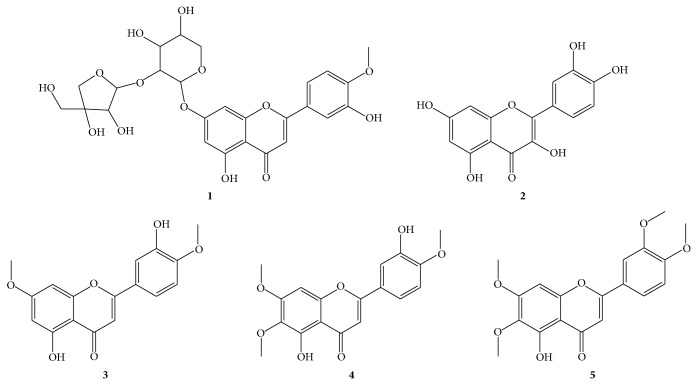
Identified compounds in hydroethanolic extract of* B. trimera *by LC-DAD-ESI-MS.

**Figure 3:**
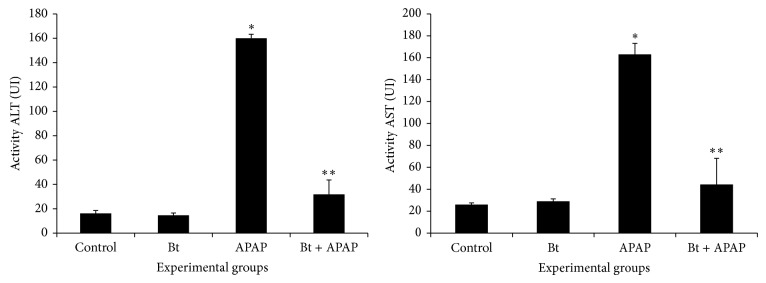
Effect of* B. trimera* hydroethanolic extract on ALT and AST activity in serum of rats 24 h after treatment with APAP. The rats were treated with 600 mg/kg* B. trimera* 1 h before administration of 835 mg/kg APAP. The data are expressed as the mean ± SD (*n* = 8). ^*^ *P* < 0.05 compared to the control, ^**^ *P* < 0.05 compared to APAP.

**Figure 4:**
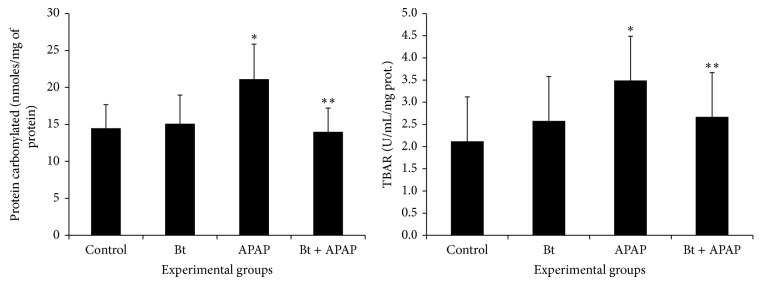
Effect of* B. trimera* hydroethanolic extract on the level of carbonylated protein and TBARS in the livers of rats 24 h after treatment with APAP. The rats were treated with 600 mg/kg* B. trimera* 1 h before administration of 835 mg/kg APAP. The data are expressed as the mean ± SD (*n* = 8). ^*^ *P* < 0.05 compared to the control, ^**^ *P* < 0.05 compared to APAP.

**Figure 5:**
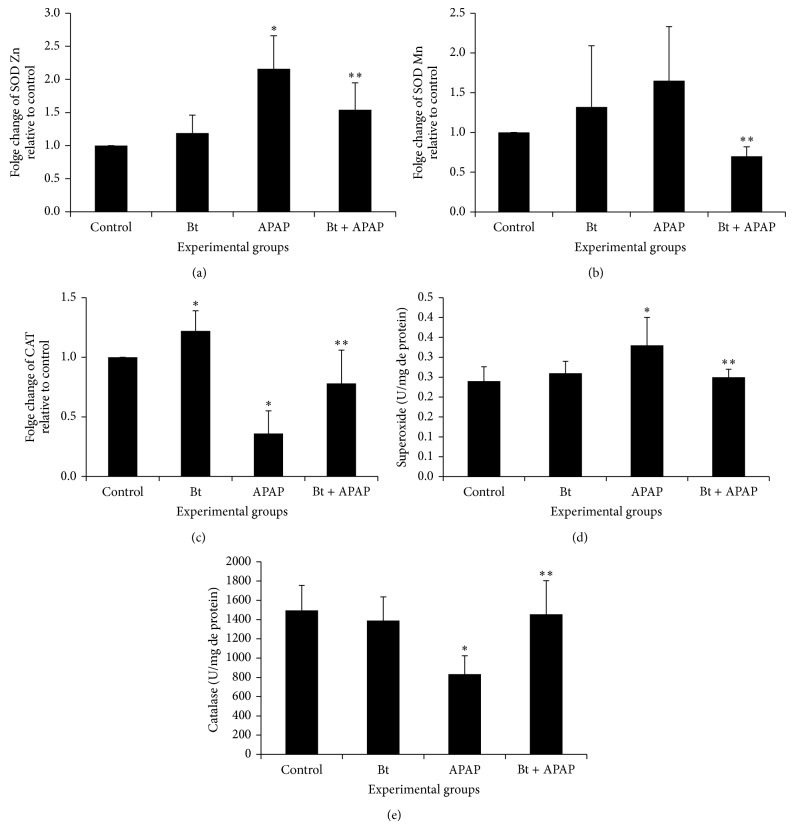
Effect of* B. trimera* hydroethanolic extract on the mRNA expression of the enzymes Zn-SOD (a), Mn-SOD (b), and CAT (c) and on the SOD (d) and CAT (e) activity in the livers of rats 24 h after treatment with APAP. The rats were treated with 600 mg/kg* B. trimera* 1 h before administration of 835 mg/kg APAP. The data are expressed as the mean ± SD (*n* = 8). ^*^ *P* < 0.05 compared to the control, ^**^ *P* < 0.05 compared to APAP.

**Figure 6:**
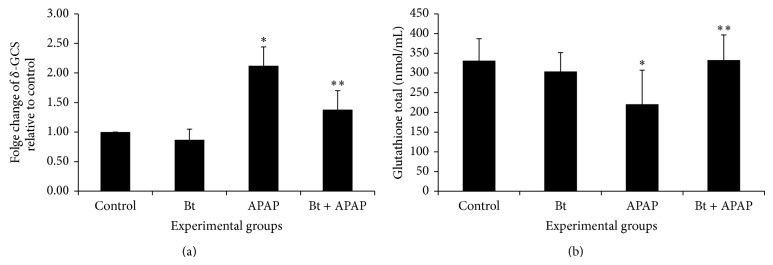
Effect of* B. trimera* hydroethanolic extract on mRNA expression of *δ*-GCS (a) and on total glutathione in rat livers 24 h after treatment with APAP (b). The rats were treated with 600 mg/kg* B. trimera* 1 h before administration of 835 mg/kg APAP. The data are expressed as the mean ± SD (*n* = 8). ^*^ *P* < 0.05 compared to the control, ^**^ *P* < 0.05 compared to APAP.

**Figure 7:**
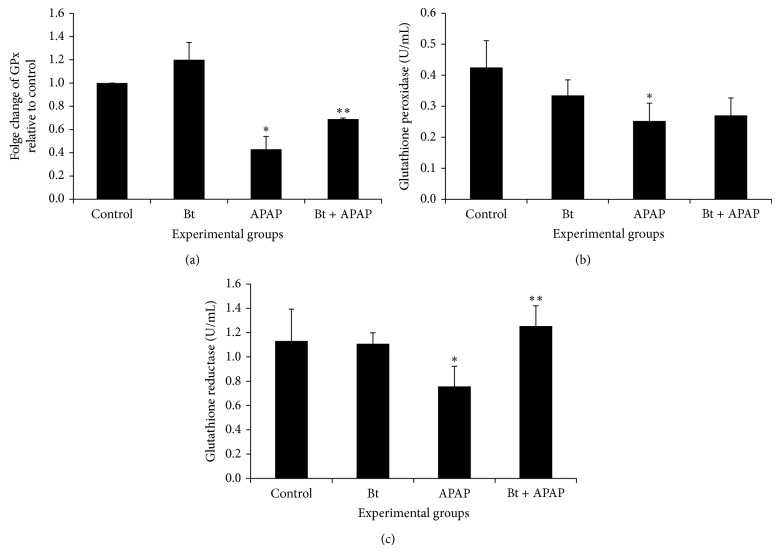
Effect of* B. trimera* hydroethanolic extract on mRNA expression of GPx (a) and on the activity of glutathione peroxidase (GPx) (b) and activity of glutathione reductase (c) in the livers of rats 24 h after treatment with APAP. The rats were treated with 600 mg/kg* B. trimera* 1 h before administration of 835 mg/kg APAP. The data are expressed as the mean ± SD (*n* = 8). ^*^ *P* < 0.05 compared to the control, ^**^ *P* < 0.05 compared to APAP.

**Figure 8:**
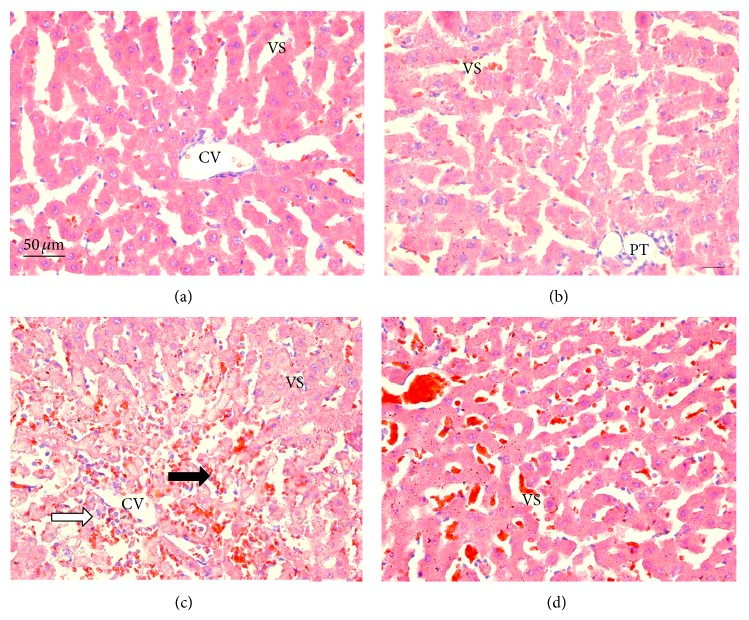
Sections of the livers of rats 24 hours after administration of PBS (a), extract of* B. trimera* (b), APAP (c), and* B. trimera* extract and APAP (d), showing (a) normal rats liver with no significant hepatic abnormalities; (b) normal rat liver with no significant hepatic abnormalities; (c) hepatic lesions, hydropic degeneration, inflammation (white arrow) and hemorrhage (black arrow); (d) well-formed polygonal hepatocytes and relatively reduced hydropic degeneration. CV: central vein; VS: venous sinuses; PT: portal triads; liver sections were stained with H & E (400x).

The pharmacological effect of the hydroalcoholic extract of* B. trimera* was investigated in our lab. A LC-DAD-ESI-MS analysis of the hydroalcoholic extract revealed presence of flavonoids, flavone, and glucosides of flavone compounds (Figure 2).

Many studies have found that the hepatotoxicity induced by APAP is the result of oxidative stress, which causes alterations in mitochondrial proteins due to a depletion of glutathione, leading to an inhibition of cellular respiration with consequent cell death [33]. Because of the biological effects described for flavonoids found in the extract, this study was conducted to investigate the effect of* B. trimera* in the modulation of oxidative stress and to evaluate the preventive effect of* B. trimera* in acetaminophen-induced liver damage.

Despite the lack of studies related to the toxicity of* B. trimera*, histopathological alterations have recently been found in the livers of pregnant rats treated with a hydroethanolic extract of the plant [22]; moreover, Rodrigues et al. [21] showed that* B. trimera* produced some genotoxic and mutagenic effects after consumption of high doses of the extract.

In this context, to analyze its toxicity, hepatic HEP G2 cells were incubated in the presence or absence of* B. trimera* extract to obtain the cellular viability/toxicity relationship. After the assays, it was observed that the hydroalcoholic extract did not present significant toxicity, and cells maintained viability of 89.0% and 98.6% after incubation periods of 1 h and 24 h, respectively. These values show no statistical difference.

Based on these results, we wondered whether the* B. trimera* extract would be able to reverse the hepatotoxicity induced by APAP. To do this, initially, we analyzed the activity of the hepatic enzymes AST and ALT. Both AST and ALT are intracellular enzymes present in large amounts in the cytoplasm of hepatocytes. Lesions or destruction of hepatic cells releases these aminotransferases into the circulation [34]. Our study showed that APAP caused a significant increase in the activity of ALT and AST. However, pretreatment with the plant extract restored this activity to values similar to those of the control. High doses of APAP have been associated with higher activity of ALT and AST [35, 36]. The ability of* B. trimera* to prevent the increase in the activity of these enzymes makes it evident that its chemical constituents exert an important hepatoprotective activity.

In addition to analyzing the activities of these hepatic enzymes, the levels of products of oxidative stress have also been described to demonstrate the occurrence of oxidative damage [37]. Among these products are substances reactive to thiobarbituric acid (TBAR) and carbonylated proteins. The levels of these substances are used as markers of redox balance in terms of lipid peroxidation and protein oxidation, respectively, in hepatic cells [38].

In our results, animals intoxicated with APAP presented high levels of carbonylated proteins and TBAR. However, pretreatment with* B. trimera* extract was able to reduce the levels of these compounds to values similar to those of their respective controls. It is known that plants, because they present antioxidant constituents, are efficient in reducing the lipid peroxidation and protein oxidation induced by APAP [39, 40]. One of the explanations for this phenomenon is that these phytochemicals are able to minimize the oxidative stress in the livers of animals intoxicated with high doses of this drug [41].

Because* B. trimera* has shown such a significant effect on the development of oxidative stress products, we evaluated its effect on the expression and activity of antioxidant enzymes. The protective action of several herbal medicines and their active constituents occurs through antioxidant enzymes (e.g., SOD, CAT, GPx, and GR), which maintain the prooxidant/antioxidant balance in the body. To eliminate ROS from the cellular system, SOD and CAT function coordinately to remove superoxide radicals [1]. Our results showed that the livers of animals intoxicated with APAP presented higher expression of the SOD-Zn and SOD-Mn isoforms. However, treatment with* B. trimera* was able to regulate this expression to values similar to those of the livers of the control animals. This increase in gene expression was followed by a higher SOD enzymatic activity. Although SOD is an antioxidant enzyme, some studies have suggested that its overexpression is in fact harmful to cells [42]. The toxic effect of ROS that has been observed in many cells overexpressing SOD has been linked to elevated levels of H_2_O_2_ and oxidative damage accompanying hydroxyl radical formation [43]. The implication is that SOD upregulation results in high H_2_O_2_ turnover.

Our results showed that the liver of animals intoxicated with APAP presented lower expression of CAT. However,* B. trimera* treatment was able to regulate this expression to values similar to those of the livers of control animals. This decrease in gene expression was followed by a lower CAT enzymatic activity. CAT activity was found to be significantly decreased after a toxic APAP dose [44]. Our results were similar in that CAT activity was significantly diminished following toxic APAP insult. This would allow for the accumulation of ROS and hydrogen peroxide, which can exacerbate the hepatocellular damage initiated by NAPQI. Treatment with the* B. trimera* extract abrogated the effect of APAP and induced an increase in CAT activity. This suggests the involvement of their antioxidant constituents in facilitating the rapid and efficient consumption of reactive oxygen species generated by APAP-mediated P450 bioactivation [36].

Glutathione (GSH/GSSG) is regarded as the main redox buffer in cells. Glutathione plays an important role in the removal of ROS and protects the thiols in biomacromolecules [45]. Under normal conditions, glutathione is mainly found in its reduced form (GSH) and in much smaller amounts in its oxidized form (GSSG) [46]. Depletion of glutathione has been associated with enhanced toxicity to chemicals, including APAP [47]. The results of our present study showed that the livers of animals intoxicated with APAP, even presenting high *δ*-GCS activity, presented levels of total glutathione less than those of the livers of control animals. However, pretreatment with* B. trimera* increased the level of total glutathione in APAP-treated animals. These results suggested that* B. trimera* could exert its hepatoprotective and radical-scavenging activities by preventing the formation of free radicals originating from APAP metabolism as well as peroxidation products and enhance the antioxidant defense system. This hypothesis is supported by recent findings that demonstrate that the antioxidant and hepatoprotective activities of extract might be mediated through augmentation of antioxidant defenses and increase in free radical inhibition due to the presence of important antioxidative factors [48].

The antioxidant effects of glutathione are directly related to GPx and GR, which are key enzymes in the maintenance of redox homeostasis via protecting cells from free radical-generated toxicity [1]. Our results show a decrease in the mRNA and enzyme activity of GPx and in the activity of the enzyme GR in the livers of rats 24 h after treatment with APAP compared to control rats. GR is an enzyme that plays a critical role in oxidative stress by APAP; a decrease in its activity will lead to interruption of the cycling between GSSG and GSH and, thus, to a shortage of GSH. Although the impairment of GR activity by APAP is not well understood, at least two hypotheses have been put forth to explain this occurrence, one invoking direct action of ROS or toxic aldehydes and another ascribing the effect to the NAPQI-GSH conjugate that forms in the presence of glutathione S-transferase [49].

The low activity of GPx is one of the early consequences of a disturbance of the prooxidant/antioxidant balance in favor of the former [50]. Inhibition of GPx increases the susceptibility of hepatocytes to paracetamol toxicity, indicating that a component of paracetamol's toxic effect involves the formation of species that are detoxified by GPx enzymes [51]. Flavonoids have been demonstrated to protect against paracetamol toxicity by inhibiting lipid peroxidation and increasing glutathione concentration [52]. Hence, the ability of plant extracts to restore the loss of GPx activity is most likely due to the presence of flavonoids. The striking increase in the GPx activity of the* B. trimera*-treated group compared with the group that received only APAP may be a result of the presence of quercetin and flavones in addition to other antioxidants in the plant.

Due to the inhibition of GPx, this study concluded that hepatic cell injury was the result of an increase in the steady-state level of H_2_O_2_ and hydroperoxides [53]. While this suggestion implies that oxidative stress is the determining factor of GSH depletion, there is also evidence to support the opposite order of events; namely, ROS production follows the depletion of GSH [54]. In other words, the levels of antioxidant enzymes, if analyzed concomitantly, allow us to infer that the animals intoxicated with APAP presented high concentration of hepatic H_2_O_2_, given that high SOD activity leads to a high production of ERO, and low CAT and GPx activities prevent the H_2_O_2_ generated from being neutralized.

To confirm the hepatoprotective effect of* B. trimera* extract against the damages caused by APAP, histopathological analyses were performed. APAP-intoxicated animals treated with the* B. trimera* extract had improved histopathology compared to the APAP-intoxicated group without treatment.

**Figure 9:**
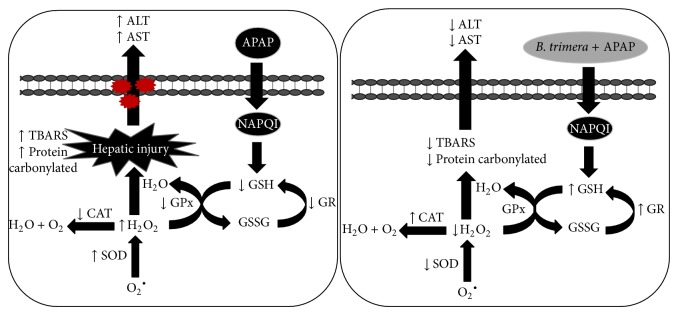
A schematic diagram showing the protective effect of* B. trimera* in acetaminophen-induced toxicity. APAP causes excessive oxidative stress and decreases the activity of antioxidant enzymes, such as GR, GPx, and CAT, and increases the activity of SOD, presumably resulting in the elevation of H_2_O_2_. The disturbance of the oxidant/antioxidant balance causes oxidative stress of the cellular system and results in hepatic injury, specifically the elevation of the ALT and AST. The administration of* B. trimera* was found to maintain the oxidant/antioxidant balance during APAP treatment, resulting in the prevention of cell damage.

In conclusion, the present study demonstrated that the hydroalcoholic extract of* B. trimera* has a hepatoprotective effect against APAP-induced hepatotoxicity in rats. The enhanced levels of antioxidant enzymes and reduced amount of peroxidation products are suggested to be the major mechanisms by which the* B. trimera* hydroalcoholic extract prevents the development of liver damage induced by APAP (Figure 9). Furthermore, a recent study published by our research group shows that the extract of* B. trimera* is also capable of modulating the activity of NADPH oxidase in peripheral neutrophils of rats intoxicated with APAP [55].

## Figures and Tables

**Table 1:** Flavonoids identified in the hydroethanolic extract of *B. trimera *by LC-DAD-ESI-MS.

Peak	Compound	RT (min)	UV (nm)	LC-MS [M − H]^−^ (*m/z*)	LC-MS [M + H]^+^ (*m/z*)
**1**	5,3′-dihydroxy-4′-methoxy-7-O-pyranosyl-furanosyl flavone	2.36	271.13; 332.13	563.49	565.38
**2**	Quercetin	3.83	255.13; 371.13	301.23	303.26
**3**	3′,5-Dihydroxy-4′,7-dimethoxyflavone	5.14	275.13; 333.13	313.32	315.28
**4**	3′,5-Dihydroxy-4′,6,7-trimethoxyflavone	5.34	274.13; 341.13	343.24	345.40
**5**	5-hydroxy-6,7,3′,4′-tetramethoxyflavone	6.07	275.13; 340.13	357.49	359.31
**6**	Unidentified	7.08	274.13	—	301.36

(—) protonated species not detected.

**Table 2:** The toxicity of the hydroethanolic extract of* B. trimera *on hepatic cells (HEP G2) and viability of cells treated with the extract. The data are expressed as a percentage. HepG2 cells were incubated in the absence of *B. trimera* extract, and Hep G2 + Cq cells correspond to the hepatic cell culture incubated with *B. trimera* extract.

Strain	1 hour		24 hours	
	Viability	Toxicity	Viability	Toxicity
HEP G2	100%	0%	100%	0%
HEP G2 + Cq	89.0%	11.0%	98.6%	1.4%
